# Effects of Active Ingredients in Alcoholic Beverages and Their De-Alcoholized Counterparts on High-Fat Diet Bees: A Comparative Study

**DOI:** 10.3390/molecules29081693

**Published:** 2024-04-09

**Authors:** Guanghe Fan, Xiaofei Wang, Cuicui Gao, Xiping Kang, Huimin Xue, Weidong Huang, Jicheng Zhan, Yilin You

**Affiliations:** 1 Beijing Key Laboratory of Viticulture and Enology, College of Food Science and Nutritional Engineering, China Agricultural University, Tsinghua East Road 17, Haidian District, Beijing 100083, China; fanguanghe@cau.edu.cn (G.F.); huanggwd@263.net (W.H.); 2 College of Food Science and Nutritional Engineering, China Agricultural University, Tsinghua East Road 17, Haidian District, Beijing 100083, China; xiaofei.wang@cau.edu.cn; 3 Xinghua Industrial Research Centre for Food Science and Human Health, China Agricultural University, Xinghua 225700, China

**Keywords:** alcoholic beverages, de-alcoholized, bioactive compounds, bees, obesity, gut microbes

## Abstract

The mechanisms by which alcohol, alcoholic beverages, and their de-alcoholized derivatives affect animal physiology, metabolism, and gut microbiota have not yet been clarified. The polyphenol, monosaccharide, amino acid, and organic acid contents of four common alcoholic beverages (Chinese Baijiu, beer, Chinese Huangjiu, and wine) and their de-alcoholized counterparts were analyzed. The research further explored how these alcoholic beverages and their non-alcoholic versions affect obesity and gut microbiota, using a high-fat diet bee model created with 2% palm oil (PO). The results showed that wine, possessing the highest polyphenol content, and its de-alcoholized form, particularly when diluted five-fold (WDX5), markedly improved the health markers of PO-fed bees, including weight, triglycerides, and total cholesterol levels in blood lymphocytes. WDX5 treatment notably increased the presence of beneficial microbes such as *Bartonella*, *Gilliamella*, and *Bifidobacterium*, while decreasing *Bombilactobacillus* abundance. Moreover, WDX5 was found to closely resemble sucrose water (SUC) in terms of gut microbial function, significantly boosting short-chain fatty acids, lipopolysaccharide metabolism, and associated enzymatic pathways, thereby favorably affecting metabolic regulation and gut microbiota stability in bees.

## 1. Introduction

Obesity is characterized by excessive accumulation of body fat and is widely recognized as a significant risk factor for chronic diseases such as diabetes, cardiovascular disease (CVD), and cancer [1]. In addition to genetic and environmental factors, obesity is also associated with an imbalance in gut microbes. Changes in gut microbes, serving as an intermediate hub for lipid metabolism and obesity development, have been strongly linked to the progression of obesity and inflammatory bowel disease [2]. The consumption of high-fat diets disrupts the balance of gut microbiota and stimulates the gene expression of microbially secreted digestive enzymes, thereby accelerating lipolysis, fat absorption, and transport, ultimately contributing to the development of obesity [3]. Weight gain is often accompanied by a decrease in the diversity of gut microbial genes and an increase in the ratio of Firmicutes to Bacteroidetes. Firmicutes, which include many known short-chain fatty acid (SCFA)-producing bacteria, particularly butyrate producers, have been associated with obesity [4]. Studies have revealed that obese adults tend to display higher concentrations of SCFAs in their stool samples when compared to lean adults [5]. High-fat diets typically reduce gut microbiota diversity, which is inversely correlated with fecal SCFA concentrations. In obese adults, SCFA concentrations are often higher, possibly due to lower efficiency in SCFA absorption and utilization [6].

The composition of gut microbiota serves as a reflection of the overall health status of the body. Bacteroidetes and Firmicutes account for up to 90% of the gut microbial population. The imbalance in the Bacteroidetes/Firmicutes ratio has been strongly linked to metabolic disorders, including obesity and inflammatory diseases [7]. Ley et al. [8] discovered a positive correlation between the Firmicutes/Bacteroidetes ratio and obesity in the human gut. Obese individuals exhibited reduced levels of Bacteroidetes, and this alteration was observed to be reversed following weight-loss intervention. The relationship between obesity and gut microbes has also been investigated in various animal models [9]. Bees provide numerous advantages as models of obesity, type 2 diabetes, neurodegenerative diseases, and immune and inflammatory diseases for studying molecular and cellular pathogenic mechanisms in human diseases [10,11,12,13,14]. The bee gut microbiota comprises nine bacterial clusters, which represent 95% to 99.9% of all individual bacteria, including *Snodgrassella alvi* of the *β-Proteobacteria*, *Gilliamella apicola* of the *γ-Proteobacteria*, *Bombilactobacillus Firm-4* and *Firm-5* in the phylum *Bombilactobacillus* of the Firmicutes phylum, and *Bifidobacterium* spp. of the genus Actinobacteria [15]. Unlike humans, mice, and other animals, bees have Firmicutes, Proteobacteria, and Actinobacteria as the main dominant phyla in their gut microbiota, which play a crucial role in maintaining metabolic homeostasis. Despite their relatively simple microbial structure and limited species diversity, the bee gut microbiota demonstrates high genomic diversity, serving as an energy reservoir that regulates host physiology and nutrient metabolism, highlighting its significance in bee health and overall ecosystem functioning [16].

Epidemiological and experimental studies have consistently established a strong association between the consumption of alcoholic beverages and the development of chronic diseases, such as cancer, CVD, diabetes, and obesity. Excessive intake of ethanol can lead to detrimental effects on various bodily systems, including damage to the central nervous system, irritation of the gastrointestinal tract, impairment of liver function, and a contribution to metabolic syndrome [17]. However, epidemiological studies have also demonstrated that moderate alcohol consumption can have beneficial effects on health, leading to improvements and a reduced risk of CVD [18]. Phenolic compounds, the primary active ingredients in alcoholic beverages such as beer, wine, and Huangjiu, have been reported to exhibit various functional effects, including improvements in glucose and lipid metabolism, protection of the cardiovascular system, and modulation of gut microbiota. Studies have demonstrated that the consumption of de-alcoholized beer can effectively ameliorate atherosclerosis in ApoE-deficient mice [19], and acute consumption of de-alcoholized beer has been shown to inhibit thrombotic activity in young adults [20]. Similarly, alcohol-free red wine has been found to provide additional cardiac protection by preventing arterial thrombosis in dietary-induced hypercholesterolemic rats [21], highlighting the potential health benefits of these phenolic compounds.

The health benefits of consuming de-alcoholized alcoholic beverages can primarily be attributed to their rich content of polyphenolic compounds. Epidemiological and clinical studies have consistently demonstrated that regular and moderate consumption of wine, typically one to two glasses per day, is associated with reduced rates of CVD, hypertension, and diabetes. Similar effects have been observed with moderate beer consumption, although to a lesser extent, likely due to the lower phenolic content in beer [22]. However, the types and levels of polyphenols in different alcoholic beverages vary considerably, influenced by their raw materials and brewing/fermentation processes. Cereal-based Chinese Baijiu (BJ) contains small amounts of phenolic acids. The total phenolic content (TPC) of Huangjiu (HJ) shows significant variation depending on the raw material source. Wu et al. [23] analyzed HJ samples with TPC ranging from 246.03 mg/L to 514.95 mg/L. Approximately 80% of the polyphenolic compounds in beer originate from barley, while 20% come from hops, with TPC ranging from 74 mg/L to 256 mg/L [24]. Polyphenols can undergo microbial conversion into more bioactive low-molecular-weight metabolites, which can have impacts on the body’s health. Furthermore, the degradation products of polyphenols have the ability to modify the composition of the gut microbiota, influencing body metabolism by promoting the growth of beneficial bacteria and inhibiting the proliferation of pathogenic bacteria [25]. Wines, known for their high polyphenol content, have been extensively studied regarding their health benefits. Resveratrol, a natural polyphenol found in wine, exhibits anti-obesity effects by protecting the intestinal barrier and modulating the composition and metabolic function of gut microbiota [26]. Grape seed proanthocyanidin has been demonstrated to reduce glucose, cholesterol, triglyceride (TG), and low-density lipoprotein (LDL) levels in obese rats [27]. Furthermore, the consumption of proanthocyanidin-rich cranberries has been found to decrease the abundance of Firmicutes and increase the abundance of Bacteroidetes in humans [28]. Therefore, consuming de-alcoholized alcoholic beverages that are rich in these bioactive compounds allows individuals to enjoy the benefits of vitamins, minerals, antioxidants, and anti-cancer agents, while avoiding the negative effects of alcohol.

In this study, a comprehensive analysis was performed on the polyphenols, monosaccharides, amino acids, and organic acids present in four popular alcoholic beverages and their de-alcoholized counterparts. Using a bee model subjected to a high-fat diet, the research assessed critical physiological and biochemical markers, including body weight, survival rates (Figure S1), and blood lipid levels. Additionally, variations in the gut microbiota across the groups were explored through 16S rRNA sequencing, providing insights into the dietary effects on microbial diversity and health implications.

## 2. Results

### 2.1. Phenols, Monosaccharides, Amino Acids, and Organic Acids in Alcoholic Beverage Samples and in Dietary Samples from Different Groups of Bees

This study primarily focused on the content of monomeric phenols (Table 1). Among the four alcoholic beverages, wine and its de-alcoholized counterpart had the highest total monomeric phenol content. The total content of 16 monomeric phenols in de-alcoholized wine was higher than that in non-de-alcoholized wine. After de-alcoholization, the levels of caffeic acid, resveratrol, and 3,4-dihydroxybenzoic acid increased, while the levels of other polyphenolic monomers decreased, with the most significant decrease observed in quercetin content. The total content of 16 monomeric phenols in de-alcoholized beer and Huangjiu was lower than that in non-de-alcoholized counterparts. Polyphenols are a class of polar compounds containing multiple hydroxyl groups. When alcoholic beverages are de-alcoholized, the change in polarity may lead to the decomposition of unstable polyphenolic substances and the generation of more stable ones, resulting in changes in the content of polyphenolic substances after de-alcoholization.

Similarly, in the diets of different bee groups, those containing wine or de-alcoholized wine had the highest total monomeric phenol content. The total content of monomeric phenols in the de-alcoholized wine additive group was lower than that in the non-de-alcoholized treatment (Table 2). After de-alcoholization, the levels of caffeic acid, resveratrol, and rutin increased, while the levels of other polyphenolic monomers decreased, with the most significant decreases observed in quercetin and catechin content. The total content of 16 monomeric phenols in de-alcoholized beer and Huangjiu additive group was lower than that in non-de-alcoholized treatment, with significant decreases observed in rutin and ferulic acid content after de-alcoholization.

### 2.2. Alcohol Tends to Exacerbate Metabolic Disorders in High-Fat-Diet-Induced Bees

**Figure 1:**
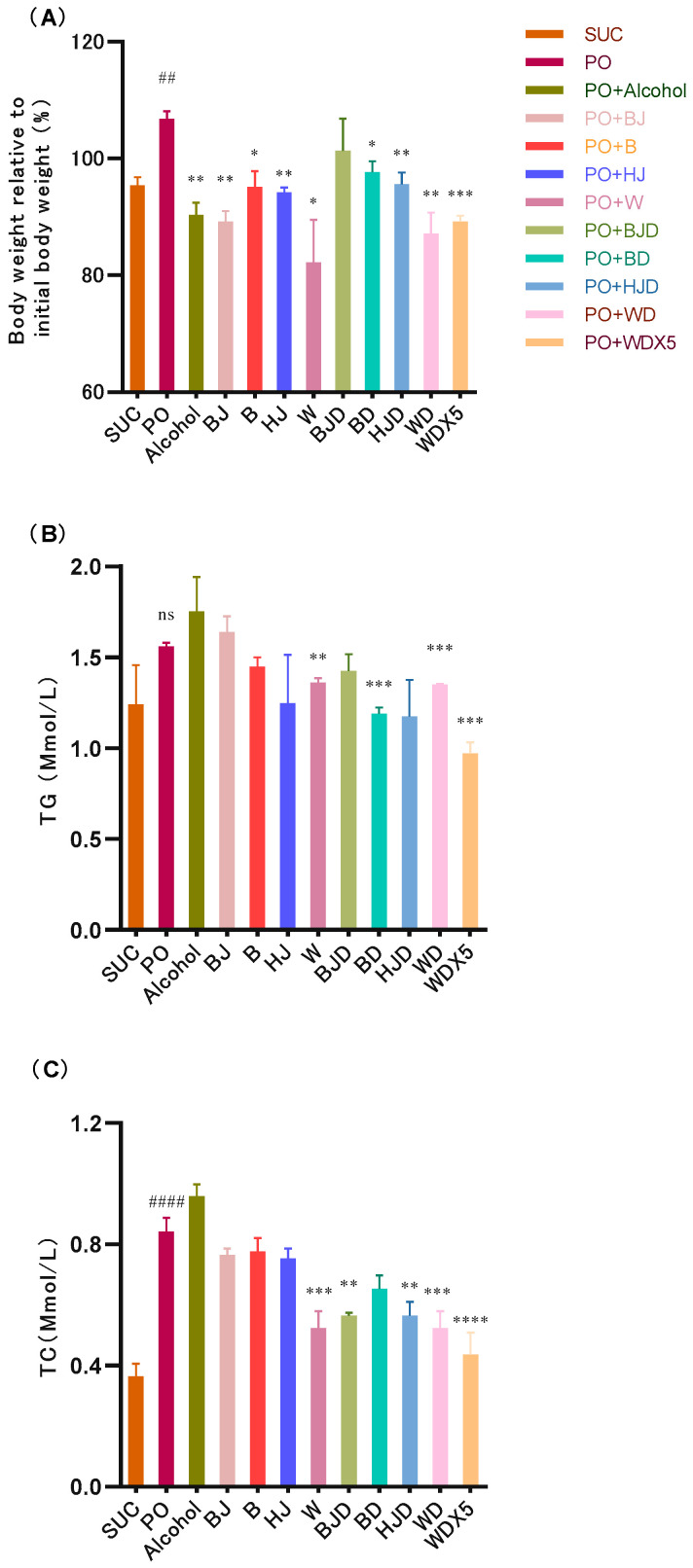
Effect of alcohol, alcoholic beverages, and their de-alcoholization treatments on the rate of body weight change (**A**) and TG (**B**) and TC (**C**) levels in PO bees. ## *p* < 0.01 and #### *p* < 0.0001 (compared with SUC) and * *p* < 0.05, ** *p* < 0.01, *** *p* < 0.001, and **** *p* < 0.0001 (compared with PO). SUC (sucrose water); PO (palm oil); BJ (Chinese Baijiu); B (beer); HJ (Chinese Huangjiu); W (wine); BJD (de-alcoholized Baijiu); BD (de-alcoholized beer); HJD (de-alcoholized Huangjiu); WD (de-alcoholized wine); WDX5 (quintuple dilution of de-alcoholized wine); alcohol (ethanol diluted in distilled water).

To investigate the impact of alcohol on host metabolism, a 2% PO diet was used to induce metabolic disorder in bees, and PO bees were treated with 1% alcohol. The results are presented in Figure 1A,B. The SUC group exhibited a decrease in body weight of approximately 4% compared to the original weight. As expected, PO bees showed a significant increase in the rate of body weight change (*p* < 0.01) (Figure 1A), with an approximate 7% increase compared to the original weight. However, the intervention with alcohol resulted in a significant decrease in the body weight of bees (approximately 10% reduction) compared to PO bees (*p* < 0.01). Regarding lipid metabolism, PO led to an increase in host TG content, although the difference was not statistically significant (Figure 1B). Furthermore, compared to the SUC group, PO significantly increased the host’s total cholesterol (TC) content (*p* < 0.01), and this increase continued after alcohol intervention.

These findings suggest that a high-fat diet can induce metabolic disorders in bees. Although alcohol treatment reduced the body weight of the bees, the results related to lipid metabolism indicate that alcohol treatment exacerbated the metabolic disorder in PO bees.

### 2.3. Alcoholic Beverages and Their Components Alleviate Metabolic Disorders in High-Fat-Diet-Induced Bees

The effects of four different alcoholic beverages on the metabolism of bees fed a high-fat diet were investigated. As depicted in Figure 1A, all four interventions with alcoholic beverages resulted in a reduction in the body weight of bees compared to the PO group. The rate of body weight change was as follows: Chinese Baijiu (BJ) group (11% decrease), beer (B) group (5% decrease), Huangjiu (HJ) group (5% decrease), and wine (W) group (20% decrease). It is notable that among all the alcoholic beverages tested, wine intervention showed the most significant trend in weight reduction and a more pronounced effect in lowering TG levels (Figure 1B). Interestingly, alcohol treatment tended to increase the TC levels in PO bees. Among the alcoholic beverages, the wine intervention, which exhibited the highest TPC (Figure S2), significantly reduced the TC levels in PO bees (*p* < 0.05). These findings suggest that alcoholic beverages rich in polyphenols have the potential to improve the metabolism of bees on a high-fat diet. Wine, with its high polyphenol content, demonstrated the most significant effects on body weight reduction and improvement of lipid profiles.

### 2.4. Consuming Non-Alcoholic Alcoholic Beverages Can Help Avoid the Adverse Effects of Alcohol While Improving Metabolic Disruptions

Consuming de-alcoholized alcoholic beverages allows you to enjoy the bioactive compounds found in alcoholic beverages while avoiding alcohol intake. In the case of beer, Huangjiu, and wine, which are rich in polyphenols, the treatment with their de-alcoholized counterparts resulted in a significant reduction in the body weight of PO bees (*p* < 0.01) (Figure 1A), with de-alcoholized wine demonstrating the strongest effect; this improvement can be attributed to the highest concentration of total phenols observed in the diets of bees fed with de-alcoholized wine (Figure S2).

Compared to alcoholic beverage treatments, the de-alcoholized beverages generally reduced TG levels. Specifically, BD, WD, and WDX5 showed significant differences (*p* < 0.01) (Figure 1B). Furthermore, while the 1% alcohol beverages decreased TC levels in PO bees, the de-alcoholized alcoholic beverage treatment further enhanced this effect, with the most significant improvement observed with WDX5. Additionally, this study found that WDX5 exhibited similar physiological indicators to the SUC group (Figure 1C). These results highlight the positive role of de-alcoholized alcoholic beverages rich in phenols in improving metabolism, with the highest content yielding the greatest improvement. Interestingly, WDX5 outperformed WD, suggesting that the nutritional components of WDX5 better matched the tolerance range of bee physiology.

### 2.5. Wine Balances the Proportions of Gut Microbiota

**Figure 2:**
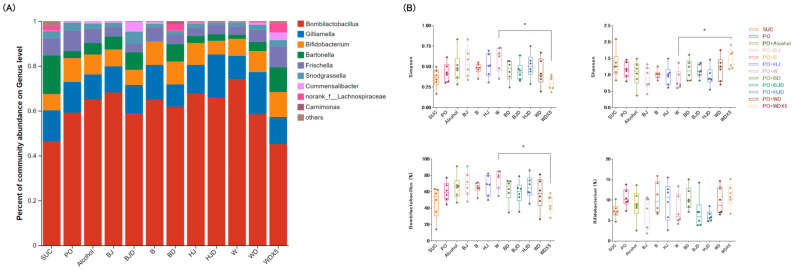
WDX5 balances the proportions of gut microbiota. (**A**) Microbial composition at the genus level. (**B**) Alpha diversity at the genus level was estimated by Shannon and Simpson indices. Relative abundance of *Bombilactobacillus* and *Bifidobacterium* in the bee gut * *p* < 0.05.

PCR amplification results confirmed that the V3-V4 variable region of the 16S rRNA gene was suitable and consistent with the experimental design (Figure S3). The predominant gut microbes identified in each group included Firmicutes, Proteobacteria, and Actinobacteria. In the assay conducted, the effects of various diets on the gut microbial composition of bees were analyzed, with a focus on examining the differences in microbial genus levels across different groups. Although there were significant differences among the groups, *Bombilactobacillus*, *Gilliamella*, *Bifidobacterium*, and *Bartonella* were found to be the dominant bacteria in the bee gut (Figure 2A). In terms of the Shannon index, both the alcohol and four alcoholic beverage treatment groups tended to decrease gut microbial diversity compared to the PO group. Although HJD had lower α diversity than the HJ treatment, the WDX5 treatment mitigated the reduction in microbial diversity caused by the high-fat diet (Figure 2B). The opposite trend observed in the Simpson index ultimately reflects the same result. *Bombilactobacillus*, which belongs to the Firmicutes phylum, is a dominant genus. Similar to previous studies, this study showed that a high-fat diet led to an increased abundance of *Bombilactobacillus*, and this trend was further enhanced by both alcohol and the four alcoholic beverage treatments. However, the corresponding de-alcoholized alcoholic beverage treatment groups exhibited a reduction in the relative abundance of *Bombilactobacillus*. Among them, WDX5 showed the most significant decrease, bringing *Bombilactobacillus* levels closer to those observed in the SUC group. In summary, alcohol consumption increased the relative abundance of *Bombilactobacillus*, but the active ingredients present in alcoholic beverages could alleviate this trend.

**Figure 3:**
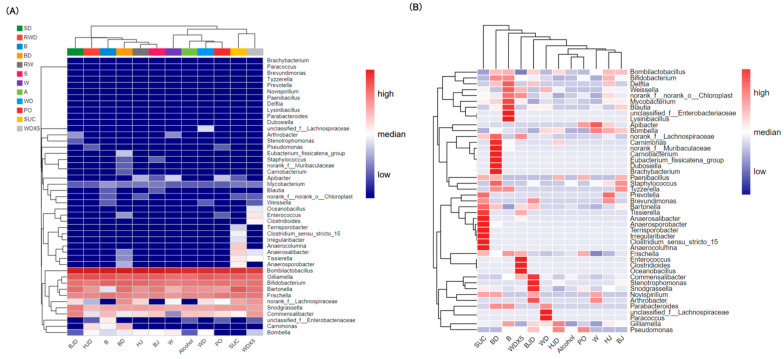
(**A**,**B**) Heatmap showing the relative abundance of genus-level bacteria within groups and among groups.

Heatmaps illustrated the differences in gut microbiota at the genus level within and between groups (Figure 3A,B). The gut microbial composition of the SUC and WDX5 groups displayed a higher degree of similarity (Figure 3A). At the absolute abundance level (Figure 3B), both the SUC and WDX5 groups exhibited lower levels of *Bombilactobacillus* compared to the other groups. The active ingredients in WDX5 appeared to ameliorate the disruption of the gut microbiota in PO bees.

### 2.6. WDX5 Improved Metabolic Function

**Figure 4:**
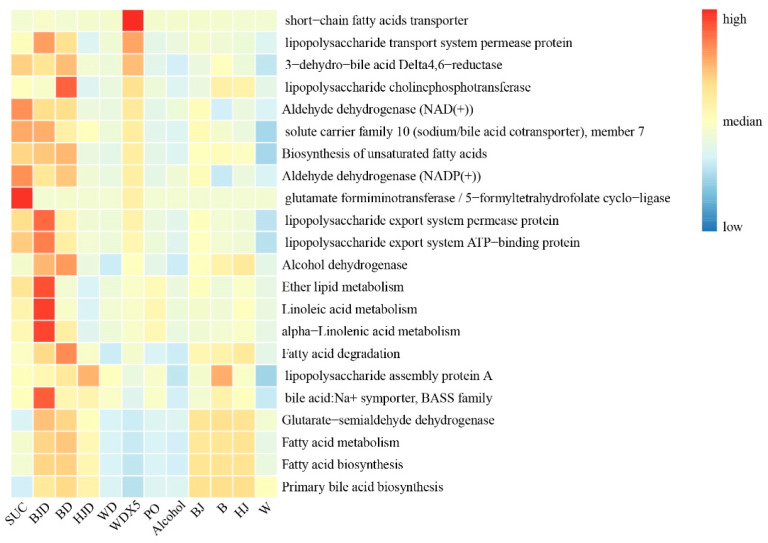
Heatmap showing differentially enriched KEGG pathway predictions of PICRUSt in bees.

In this study, Picrust 2 was employed to predict the functional profiles of representative sequences from the gut microbiota. As depicted in Figure 4, different bee diets resulted in significant alterations in the functional capacities of the bee gut microbiota. The gut microbiota of bees in the SUC group exhibited functional advantages in glutamate and lipopolysaccharide metabolism. However, both the PO and alcohol treatments generally reduced the functional potential of the bee gut microbiota in fatty acid, glutamate, and lipopolysaccharide metabolism. These findings suggest that a high-fat diet and alcohol consumption can significantly impact the metabolic functions of the bee gut microbiota. In comparison to the PO and alcohol treatments, the four alcoholic beverage treatment groups exhibited a slight improvement in lipopolysaccharide and SCFA metabolism, although the effects were not prominent. Nevertheless, the four alcoholic beverages demonstrated superior functional effects in alcohol metabolism and SCFA metabolism. Furthermore, the de-alcoholization of all four alcoholic beverages significantly enhanced the metabolic functions associated with lipopolysaccharide metabolism, SCFA metabolism, alcohol metabolism, and fatty acid degradation. Notably, WDX5 treatment exhibited a more pronounced enhancement in metabolic pathways compared to the other de-alcoholized alcoholic beverage treatment groups.

## 3. Discussion

In nature, bees primarily consume pollen as their main source of oil intake. Pollen is similar in composition to palm oil, which consists mainly of palmitic, oleic, linoleic, and myristic acids. Bees are capable of effectively digesting and absorbing palm oil. In this study, bees were treated with a 2% palm oil diet to induce metabolic disorders. The results revealed a significant increase in body weight, as well as elevated levels of TC and TG in the hemolymph of PO bees compared to those on the SUC diet. This high-lipid induction disrupts the homeostasis of body lipid metabolism, leading to lipid accumulation and subsequent weight gain. In the study conducted by Wang et al. [29], they observed that excessive intake of dietary fat, particularly palm oil, resulted in increased bee body weight, decreased survival rate, and fat accumulation, which is consistent with our research findings.

Alcohol intake affects glucolipid metabolism, reduces superoxide dismutase activity, increases malondialdehyde levels, and disrupts the balance between oxidative and antioxidant activity, leading to lipid peroxidation [30]. The PO bees experienced weight gain, while the alcohol-treated group exhibited significant weight loss compared to the SUC group. Both conditions significantly reduced the survival rate, with the alcohol-treated group demonstrating a lower survival rate. An analysis of the nutrient composition of the SUC, alcohol, and PO revealed a high concentration of monosaccharides. The PO disrupted the bees’ glycolipid metabolism and increased TG and TC levels. Additionally, the PO group contained toxic substances such as rhamnose, mannose, and galactose, which negatively impact the bees and shorten their lifespan [31]. Furthermore, the addition of alcohol aggravated the metabolic disorders in bees on a high-fat diet, increasing TG and TC levels and further reducing their survival rate.

Moreover, this paper is dedicated to comparing the impact of different alcoholic beverages on bees. Baijiu, which contained minimal nutrients such as glucose, lactic acid, acetic acid, and citric acid, showed similar effects to alcohol in terms of body weight changes, survival rate, and lipid alterations. Similarly, the minimal nutrient content in BJD resulted in comparable outcomes to bees on a high-fat diet in terms of body weight, survival rate, and blood lipid changes.

Plant extracts rich in polyphenols have shown positive effects in improving plasma lipid levels and reducing lipid accumulation in animal models fed high-fat, high-glucose diets [32]. HJ, which contained a small amount of ferulic acid along with glucose, lactic acid, and citric acid, exhibited a significant weight-loss trend and slower death rate compared to the PO group. HJD, which had more weight loss and higher survival rates while showing relatively lower levels of TG and TC, may be attributed to the regulation of polyphenolic active ingredients. Studies have shown that rutin positively affects gut microbiota disorders and metabolic disorders associated with obesity in obese individuals [33]. BD contained significant amounts of rutin, ferulic acid, and erucic acid. Although the beer treatment led to more weight loss compared to the BD group, BD exhibited a higher survival rate. WD and WDX5 significantly reduced bee body weight, with WD showing greater weight loss than compared to WDX5. The WD and WDX5 groups had higher survival rates and lower relative levels of TG and TC. WD and WDX5 were rich in gallic acid, catechins, quercetin, rutin, vanillic acid, resveratrol, and ferulic acid. Some of these compounds, including catechin, resveratrol, ferulic acid, and gallic acid, play important roles in alleviating obesity and improving lipid metabolism capacity [34]. The weight loss and mortality tendency in the WD and WDX5 groups were significantly reduced, suggesting the regulatory effects of polyphenolic active ingredients. Interestingly, there were no significant differences in amino acid and polysaccharide composition between the WD and WDX5 groups. However, the content of polyphenols and organic acids in WD was approximately five times higher than that in WDX5. WDX5 demonstrated advantages in terms of survival rate and regulation of lipid metabolism, potentially indicating that exceeding the acceptable range of polyphenol and organic acid content in bees could lead to side effects. This paper focuses exclusively on the health effects of polyphenolic nutrients found in alcoholic beverages; although alcoholic beverages also contain oligosaccharides and peptides, this study does not delve into their health implications. However, it opens the door for future research to explore these aspects further.

The PO bees exhibited increased body weight and reduced abundance and diversity in their gut microbiota. PICRUSt analysis revealed that PO inhibited pathways related to SCFA, glutamate, and lipopolysaccharide metabolism, while alcohol tended to enhance these pathways. The WDX5 treatment showed enhanced lipid-metabolism-related pathways and played a beneficial role in regulating lipid metabolism disorders in bees. *Bartonella* was found to dominate the gut microbiota of winter bees and likely plays a significant role in energy metabolism regulation [35]. Both SUC and WDX5 treatments showed a higher abundance of *Bartonella*. Alcoholic beverage treatment increased the abundance of *Bombilactobacillus* and decreased *Bartonella* levels. De-alcoholized alcoholic beverage treatment reduced the growth trend of *Bombilactobacillus*, resulting in a more balanced gut microbiota structure. Notably, the WDX5 treatment group increased the abundance of *Bartonella*, *Gilliamella*, and *Bifidobacterium*, while reducing *Bombilactobacillus* levels, making its gut microbiota function most similar to that of the SUC group. Contrary to findings in mice, Júnior et al. [36] found that a high-fat and high-sugar diet increased the relative abundance of Firmicutes and Actinobacteria, while decreasing Bacteroidetes and *Bombilactobacillus* levels; the present study observed an increase in *Bombilactobacillus* in PO bees and a subsequent decrease after WDX5 intervention. In another study, mice fed a high-fat diet exhibited higher levels of Proteobacteria and Firmicutes compared to those on a normal diet. While Firmicutes were also higher in PO bees than in the SUC group, Proteobacteria showed opposite results [37].

These results highlight the moderating effect of de-alcoholized wine treatment on PO bees, likely attributed to its polyphenolic composition. Although WD contained higher levels of active ingredients than WDX5, the superior effect of WDX5 may be due to its polyphenol concentration being more suitable for bees, emphasizing the importance of a balanced nutritional intake. Moderate consumption of red wine has been shown to have a notable effect on the growth of specific gut microbiota, promoting overall health [38], and non-ethanol components in alcoholic beverages significantly affect the diversity of host gut bacteria and metabolic composition, further impacting host health [39]. Moreover, common findings obtained by the three approaches (in vitro, animal models, and human nutritional interventions) such as the fact that the Firmicutes/Bacteroidetes ratio tends to decrease after the feed/intake/consumption of grape/wine polyphenols are highlighted [40]. However, this study predominantly involves the analysis of the obtained results. Future research will build upon the findings of this study, with a particular focus on the pathways of lipid metabolism, aiming to explore the interplay between changes in the gut microbiota and metabolic disruptions at the molecular level. In summary, there is a strong correlation between the bioactive compounds in alcoholic beverages and the improvement of physiological markers and gut microbiota in high-frequency foraging bees. In conclusion, a strong correlation exists between the active compounds present in alcoholic beverages and the improvement of physiological indicators and the gut microbiota in PO bees.

## 4. Materials and Methods

### 4.1. De-Alcoholization of Alcoholic Beverages

The wine originated from Huailai regions of China and was produced in the year 2016, and the Huangjiu, beer, and Baijiu were procured from the Chinese market. The wine, Huangjiu, beer, and Baijiu had alcohol contents of 13%, 3.6%, 17%, and 53%, respectively. Using a rotary evaporator set at 40 °C and 90 rotations per minute (r/min), 50 mL samples of wine, Huangjiu, beer, and Baijiu were subjected to de-alcoholization treatment. The distillation process was ceased once no further distillate was obtained. The remaining solution, representing the de-alcoholized form of the alcoholic beverages, was collected in a centrifuge tube. Ultra-pure water was added to the collected solution, and the final volume was adjusted to 50 mL using a volumetric flask.

### 4.2. Alcoholic Beverages and Their De-Alcoholized Counterparts and Bee Diet Composition Analysis

The determination of TPC was carried out using the Folin–Ciocalteu colorimetric method [41]. Gallic acid standard solutions with gradient concentrations were prepared. From these, 1 mL of each concentration was added to a 25 mL volumetric flask, followed by the incorporation of 3 mL of Folin–Ciocalteu reagent, ensuring thorough mixing. Next, 6 mL of 12% sodium carbonate solution was added, and the final volume was adjusted to 25 mL. Employing this protocol, the total phenolic content in alcoholic beverages and their de-alcoholized counterparts was similarly assessed. The absorbance of the mixture was measured at 765 nm using a UV-1800 spectrophotometer (Shimadzu, Japan). The TPC values were expressed as milligrams of gallic acid per milliliter of sample (mg/L). All measurements were performed in triplicate to ensure accuracy and reliability.

The quantification of 16 monomer polyphenols in the samples was performed using a Waters Acquity I-Class UPLC system equipped with a BEH C18 column (2.1 × 100 mm, 1.7 μm) and coupled to a Xevo TQ-S mass spectrometer (Waters, Milford, MA, USA). The column was maintained at 30 °C throughout the analysis. A 3 μL injection volume and a flow rate of 0.3 mL/min were employed. The separation of the target compounds was achieved by manipulating the composition of the mobile phase. Initially, the mobile phase consisted of 90% solvent A, which is composed of 0.1% formic acid in water, and 10% solvent B, containing 0.1% formic acid in methanol. This initial condition was maintained for the first 4–5 min of the chromatographic run. Subsequently, a gradual transition was made to alter the composition of the mobile phase. The solvent conditions were adjusted to 60% A and 40% B at the 4–5 min mark, which was followed by a further shift to 40% A and 60% B at the 6 min point in the analysis. Finally, at the 8 min mark, the chromatography returned to its original conditions, with 90% A and 10% B. Before UPLC analysis, all samples were filtered through 0.22 μm organic filters. The methods for the detection of monosaccharides, amino acids, and organic acids have been detailed in the Supplementary Materials.

### 4.3. Animal Experiments

**Figure 5:**
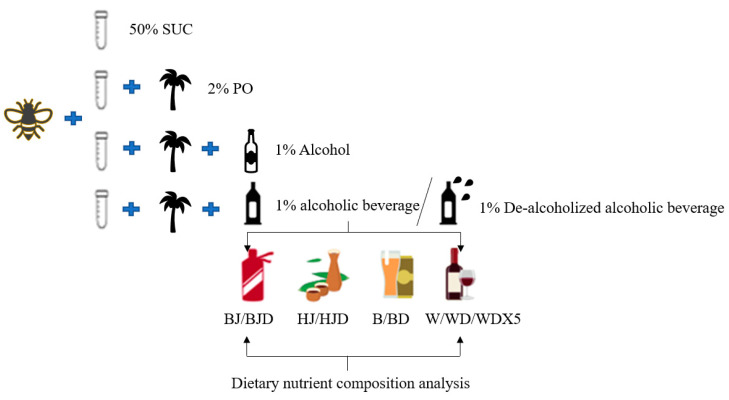
Schematic representation of bee grouping. SUC (sucrose water); PO (palm oil); BJ (Chinese Baijiu); B (beer); HJ (Chinese Huangjiu); W (wine); BJD (de-alcoholized Baijiu); BD (de-alcoholized beer); HJD (de-alcoholized Huangjiu); WD (de-alcoholized wine); WDX5 (quintuple dilution of de-alcoholized wine); alcohol (ethanol diluted in distilled water).

The bees used in this study were obtained from Xinghua, Jiangsu Province, and were reared in a controlled environment. They were housed in a constant-temperature incubator set at 35 °C with a relative humidity of 65% [42]. Bees were cultured in 400 mL covered plastic cups, with each culturing unit containing 20 bees. The experiment was conducted with three replicates per treatment. To maintain the optimal conditions for the bees, their diets were monitored every 2 days during the 12-day incubation period, guaranteeing a consistent and sufficient supply of essential nutrients. Bee mortality was recorded at regular intervals each day, and any dead bees were promptly removed from the culture to maintain a healthy and controlled environment. Additionally, the weight of each bee culture unit was measured every three days using the spatial stress method to assess the impact of the treatments on bee development and health. In order to investigate the impact of alcoholic beverages on PO bees, an improved version of the method described by Wang [29] was employed. Specifically, a high-fat diet was induced using 2% palm oil (PO). The experimental design consisted of different groups, as illustrated in Figure 5.

One group of bees was provided with a normal diet consisting of 50% sucrose water (SUC), while the remaining bees were fed a high-fat diet comprising 50% sucrose water and 2% palm oil (PO). The PO group was further divided into eleven treatment groups as outlined in Table S2. These included 1% alcohol treatment group (alcohol), 1% alcoholic beverage treatment groups (Chinese Baijiu, beer, Huangjiu, and wine treatment groups: BJ, B, HJ, and W, respectively), and 1% de-alcoholized alcoholic beverage treatment groups (Baijiu, beer, Huangjiu, and wine, denoted as BJD, BD, HJD, and WD, respectively). Additionally, the de-alcoholized wine was diluted five-fold and represented by the treatment group WDX5.

### 4.4. Determination of Hemolymph TG and TC in Bees

Briefly, the hemolymph of each bee was removed by making a small incision at the neck and carefully collecting it using a 10 µL pipette. Great care was taken to prevent any contamination from the intestinal hemolymph, and the collected hemolymph samples were immediately frozen at −80 °C until analysis. TG and TC levels in the bee hemolymph were determined by using the TG (A110-1-1) and TC (A111-1-1) detection kits of Nanjing Jiancheng Bioengineering Institute (Nanjing, China).

### 4.5. Gut Microbiota Analysis by 16SrRNA Sequencing

At the end of the bee culture period, the gut microbiota samples of six bees in each treatment group were randomly extracted on an ultra-clean bench and stored in a 1.5 mL centrifuge tube in the refrigerator at −80 °C. Total DNA was extracted from the microflora according to the E.Z.N.A.^®^ soil DNA kit (Omega Bio-tek, Norcross, GA, USA), and the quality of the extracted DNA was assessed through 1% agarose gel electrophoresis, while the purity and concentration of the DNA were measured using NanoDrop2000 (Thermo Fisher Scientific, Waltham, MA, USA). 16S rRNA genes of V3–V4 regions were amplified using specific primers (515F: 5′-GTGCCAGCMGCCGCGGTAA-3′ and 806R: 5′-GGACTACHVGGGTWTCTAAT-3′). All PCR reaction mixtures were composed as follows: 4 μL of 5× TransStart FastPfu buffer, 2 μL of 2.5 mM dNTPs, 0.8 μL of upstream and 0.8 μL of downstream primers (each at 5 μM), 0.4 μL of TransStart FastPfu DNA polymerase, and 10 ng of template DNA. The total volume was brought up to 20 μL with the addition of nuclease-free water. Amplification was performed as follows: predenaturation at 95 °C for 3 min, followed by 27 cycles of denaturation at 95 °C for 30 s, annealing at 55 °C for 30 s, and extension at 72 °C for 45 s, followed by stable extension at 72 °C for 10 min, and finally storage at 10 °C. PCR products were verified by 2% agarose gel electrophoresis, and the recovered products were purified using the AxyPrep DNA Gel Extraction Kit (Axygen Biosciences, Union City, CA, USA). The final libraries obtained from the PCR products were sequenced using Illumina’s ABI GeneAmp^®^ 9700 (Illumina, USA). Bioinformatics analysis of the sequences involved OTU clustering and removal of chimeric sequences based on a 97% similarity threshold using UPARSE software [43] (http://drive5.com/uparse/, version 7.1; accessed on 12 March 2021). Each sequence was annotated for species classification using the ribosomal database project (version 11.4) classifier with a comparison threshold set at 70% [44]. The Silva 16S rRNA database (v138) was used for sequence alignment. Functional predictions of the gut microbiota were conducted using PICRUSt, and alpha and beta diversity analyses were performed to assess the species richness and evenness of the gut microbiota.

### 4.6. Statistical Analysis

All results are presented as means ± SEM (standard error of the mean) and were calculated using GraphPad Prism 8.0 software. Significance levels for all statistical analyses were set at *p* < 0.05. The differences between groups were analyzed using one-way analysis of variance (ANOVA). Heatmaps were generated in R programming language using the heatmap package [45].

## Figures and Tables

**Table 1:** Concentration of phenols, monosaccharides, amino acids, and organic acids in alcoholic beverages and their de-alcoholized counterpart samples (mg/L).

Composition	W	WD	B	BD	HJ	HJD	BJ	BJD
Gallic acid	273.0 ± 0.9	301.0 ± 0.2	—	—	—	—	—	—
Caffeic acid	5.0 ± 0.2	6 ± 0.4	—	—	—	—	—	—
Catechin	72.0. ± 0.8	63 ± 0.7	—	—	—	—	—	—
L-Epicatechin	6.0 ± 0.1	5.0 ± 0.1	—	—	—	—	—	—
Quercetin	95.0 ± 0.7	7.0 ± 0.2	—	—	—	—	—	—
Chlorogenic acid	0.6 ± 0.1	1.0 ± 0.1	—	—	—	—	—	—
(-)-Gallocatechin	—	—	10.0 ± 0.2	1.0 ± 0.1	—	—		
Rutin	46.0 ± 0.2	64.0 ± 0.9	33.0 ± 0.3	27.0 ± 0.7	—	—	—	—
Vanillic acid	9.0 ± 0.7	10.5 ± 0.7	—	—	—	—	—	—
Resveratrol	24.0 ± 0.2	30.0 ± 0.2	—	—	—	—	—	—
Kaempferol	1.0 ± 0.1	0.40 ± 0.01	—	—	—	—	—	—
Ferulic acid	2.0 ± 0.1	2.0 ± 0.1	73.0 ± 0.3	63.0 ± 0.5	47.0 ± 0.5	36.0 ± 0.9	—	—
Para-hydroxybenzoic acid	0.60 ± 0.03	0.60 ± 0.09	—	—	—	—	—	—
Erucic acid	4.0 ± 0.1	1.0 ± 0.1	10.0 ± 0.2	9.0 ± 0.3	0.50 ± 0.02	0.50 ± 0.02	—	—
3,4-Dihydroxybenzoic acid	22 ± 0.34	24 ± 0.39	0.1 ± 0.03	0.1 ± 0.03	—	—	—	—
Total monomeric phenolics	476.0 ± 0.8	518.0 ± 0.9	117.0 ± 0.4	99.0 ± 0.7	48.0 ± 0.2	37.0 ± 0.8	—	—
Mannose	280.0 ± 0.4	301.0 ± 0.3	9.0 ± 0.6	74.0 ± 0.1	8.0 ± 0.7	301.0 ± 0.1	—	—
Ribose	82.0 ± 0.4	79.0 ± 0.5	—	172.0 ± 0.1	—	41.0 ± 0.3	—	—
Rhamnose	243.0 ± 0.4	241.0 ± 0.1	—	—	—	—	—	—
Glucuronic acid	44.0 ± 0.1	48.0 ± 0.7	399.0 ± 0.3	506.0 ± 0.1	361.0 ± 0.4	560.0 ± 0.1	—	—
Galacturonic acid	406.0. ± 0.7	410.0 ± 0.5	8 ± 0.1	—	—	—	—	—
Glucose	1000.0 ± 0.9	1015.0 ± 0.6	20,847.0 ± 0.1	27,026.0 ± 0.1	18,784.0 ± 0.3	34,746.0 ± 0.4	3.0 ± 0.2	6.0 ± 0.1
Galactose	214.0 ± 0.1	219.0 ± 0.7	—	40.0 ± 0.4	—	100.0 ± 0.6	—	—
Xylose	135.0 ± 0.7	141.0 ± 0.3	—	233.0 ± 0.1	—	284.0 ± 0.1	1.0 ± 0.4	1.0. ± 0.5
Arabinose	214.0 ± 0.1	220.0 ± 0.3	—	140.0 ± 0.4	—	180.0 ± 0.3	—	—
Fucose	25.0 ± 0.2	39.0 ± 0.1	—	—	—	166.0 ± 0.5	—	—
Total monosaccharides	2646.0 ± 0.9	2717.0 ± 0.7	21,263.0 ± 0.2	28,192.0 ± 0.6	19,155.0 ± 0.8	36,381.0 ± 0.4	4.0 ± 0.1	7.0 ± 0.5
Aspartic acid	124.0 ± 0.1	52.0 ± 0.3	279.0 ± 0.6	290.0 ± 0.7	132.0 ± 0.9	54.0 ± 0.3	—	—
Glutamic acid	444.0 ± 0.6	48.0 ± 0.8	1015.0 ± 0.9	1050.0 ± 0.7	453.0 ± 0.5	56.0 ± 0.5	—	—
Hydroxyproline	4.0 ± 0.1	11.0 ± 0.2	6.0 ± 0.3	6.0 ± 0.3	4.0 ± 0.1	11.0 ± 0.2	—	—
Serine	72.0. ± 0.5	38.0 ± 0.2	265.0 ± 0.4	264.0 ± 0.3	74.0 ± 0.1	36.0 ± 0.2	—	—
Glycine	67.0 ± 0.1	31.0 ± 0.2	293.0 ± 0.9	293.0. ± 0.9	71.0 ± 0.1	32.0 ± 0.1	—	—
Histidine	29.0 ± 0.2	3.0 ± 0.1	104.0. ± 0.2	102.0 ± 0.2	30.0 ± 0.3	3.0 ± 0.1	—	—
Arginine	50.0 ± 0.6	4.0 ± 0.1	252.0 ± 0.4	250.0 ± 0.3	52.0 ± 0.3	4.0 ± 0.5	—	—
Threonine	34.0. ± 0.3	16.0 ± 0.2	159.0 ± 0.2	164.0 ± 0.4	36.0 ± 0.3	19.0 ± 0.3	—	—
Alanine	116.0 ± 0.4	39.0. ± 0.3	362.0 ± 0.4	380.0 ± 0.5	124.0 ± 0.3	44.0 ± 0.3	—	—
Proline	252.0 ± 0.4	804.0 ± 0.8	665.0 ± 0.7	676.0 ± 0.7	276.0 ± 0.4	849.0 ± 0.6	—	—
Tyrosine	50.0 ± 0.4	7.0. ± 0.3	187.0 ± 0.5	191.0 ± 0.8	53.0 ± 0.6	8.0 ± 0.1		
Valine	67.0 ± 0.4	20.0 ± 0.3	227.0 ± 0.7	232.0 ± 0.6	68.0 ± 0.5	23.0 ± 0.4		
Methionine	8.0 ± 0.1	2.0 ± 0.1	16.0 ± 0.2	210 ± 0.2	11.0 ± 0.3	2.0 ± 0.3		
Cysteine	4.0 ± 0.1	13.0 ± 0.2	13.0 ± 0.2	60.0. ± 0.2	5.0 ± 0.2	10.0 ± 0.2		
Isoleucine	35.0 ± 0.3	18.0 ± 0.2	169.0 ± 0.4	177.0 ± 0.3	35.0 ± 0.1	20.0 ± 0.4		
Leucine	64.0. ± 0.5	32.0 ± 0.3	310.0 ± 0.6	324.0 ± 0.7	67.0 ± 0.3	49.0 ± 0.5		
Ortholeucine	—	—	—	—	—	—		
Phenylalanine	52.0 ± 0.3	18.0 ± 0.3	251.0 ± 0.7	249.0 ± 0.5	55.0 ± 0.4	19.0 ± 0.3		
Lysine	44.0 ± 0.5	18.0. ± 0.5	108.0 ± 0.7	119.0 ± 0.4	46.0 ± 0.4	22.0. ± 0.3	—	—
Total amino acids	1518.0. ± 0.9	1174.0 ± 0.8	4680.0 ± 0.4	4793.0 ± 0.4	1591.0 ± 0.5	1261.0 ± 0.4	—	—
Oxalic acid	31.0 ± 0.9	32.0 ± 0.5	3.0 ± 0.3	3.0 ± 0.1	32.0 ± 0.5	31.0 ± 0.3	0.40 ± 0.03	0.40 ± 0.07
Tartaric acid	1255.0 ± 0.7	1687.0. ± 0.8	—	—	29.0 ± 0.7	27.0. ± 0.1	—	—
Malic acid	—	—	58.0 ± 0.5	53.0 ± 0.3	14.0 ± 0.1	9.0 ± 0.01	—	—
Lactic acid	1719.0 ± 0.05	2233.0 ± 0.6	—	133.0 ± 0.1	156.0 ± 0.9	873.0. ± 0.3	4763.0 ± 0.4	1728.0 ± 0.4
Acetic acid	156.0 ± 0.5	—	2962.0 ± 0.5	—	—	58.0 ± 0.6	—	—
Maleic acid	0.20 ± 0.06	0.10 ± 0.07	10.0 ± 0.3	9.0 ± 0.5	24.0 ± 0.5	10.0 ± 0.1	—	0.30 ± 0.06
Citric acid	64.0 ± 0.6	286.0 ± 0.4	1460.0 ± 0.7	1311.0 ± 0.7	617.0 ± 0.1	3857.0 ± 0.4	5.0 ± 0.6	38.0 ± 0.8
Fumaric acid	—	—	1.80 ± 0.07	1.6 ± 0.4	0.40 ± 0.05	—	—	0.20 ± 0.02
Succinic acid	22.0 ± 0.7	3363.0 ± 0.1	12.0. ± 0.1	12.0 ± 0.5	2491.0. ± 0.7	18.0 ± 0.1	4.7 ± 0.1	91.0 ± 0.6
Total organic acids	3550.0 ± 0.7	7566.0 ± 0.2	1703.0 ± 0.02	1524.0 ± 0.5	5141.0 ± 0.6	8716.0 ± 0.5	4773.0 ± 0.2	1859.0 ± 0.3

“—” means not detected. SUC (sucrose water); PO (Palm Oil); BJ (Chinese Baijiu); B (beer); HJ (Chinese Huangjiu); W (Wine); BJD (de-alcoholized Baijiu); BD (de-alcoholized Beer); HJD (de-alcoholized Huangjiu); WD (de-alcoholized wine); WDX5 (quintuple dilution of de-alcoholized wine); alcohol (ethanol diluted in distilled water).

**Table 2:** Concentration of phenols, monosaccharides, amino acids, and organic acids in the diets of different bee groups (mg/L).

Composition	W	WD	WDX5	B	BD	HJ	HJD	BJ	BJD	SUC	Alcohol	PO
Gallic acid	44.0 ± 0.7	420 ± 0.2	9.0 ± 0.1	—	—	—	—	—	—	—	—	—
Caffeic acid	0.80 ± 0.04	0.90 ± 0.01	0.20 ± 0.02	—	—	—	—	—	—	—	—	—
Catechin	12.0 ± 0.3	9.0 ± 0.1	2.0 ± 0.1	—	—	—	—	—	—	—	—	—
Epicatechin	0.90 ± 0.09	0.70 ± 0.03	0.10 ± 0.04	—	—	—	—	—	—	—	—	—
Quercetin	1.60 ± 0.02	1.0 ± 0.1	0.20 ± 0.03	—	—	—	—	—	—	—	—	—
Chlorogenic acid	—	—	—	0.40 ± 0.08	0.40 ± 0.01	—	—	—	—	—	—	—
Rutin	8.0 ± 0.3	9.0 ± 0.1	2.0 ± 0.2	13.0 ± 0.3	10.0 ± 0.2	—	—	—	—	—	—	—
Vanillic acid	1.5 ± 0.2	1.5 ± 0.1	0.30 ± 0.02	—	—	—	—	—	—	—	—	—
Resveratrol	4.0. ± 0.1	4.0 ± 0.8	0.80 ± 0.01	—	—	—	—	—	—	—	—	—
Kaempferol	0.20 ± 0.01	0.10 ± 0.01	—	—	—	—	—	—	—	—	—	—
Ferulic acid	0.30 ± 0.04	0.20 ± 0.04	—	28.0 ± 0.9	24.0 ± 0.4	3.8 ± 0.3	2.8 ± 0.2	—	—	—	—	—
Para-hydroxybenzoic acid	0.10 ± 0.03	—	—	—	—	—	—	—	—	—	—	—
Erucic acid	0.60 ± 0.04	0.20 ± 0.01	-	3.7 ± 0.8	3.3 ± 0.1	—	—	—	—	—	—	—
3,4-Dihydroxybenzoic acid	3.7 ± 0.2	3.3 ± 0.8	0.60 ± 0.08	—	—	—	—	—	—	—	—	—
Total monomeric phenolics	78.0 ± 0.7	72.0 ± 0.9	15.0 ± 0.4	45.0 ± 0.6	38.0 ± 0.7	4.0 ± 0.6	3.0 ± 0.2	—	—	—	—	—
Mannose	10.7 ± 0.2	11.7 ± 0.1	11.6 ± 0.3	28.3 ± 0.6	21.0 ± 0.5	12.4 ± 0.2	15.0 ± 0.7	12.4 ± 0.4	11.0 ± 0.2	6140.2 ± 0.4	1313.9 ± 0.2	6080.2 ± 0.1
Ribose	61.4 ± 0.1	47.7 ± 0.4	40.3 ± 0.9	98.2 ± 0.8	64.8 ± 0.6	30.1 ± 0.5	42.4 ± 0.3	—	—	799.9 ± 0.6	369.0 ± 0.7	1247.6 ± 0.7
rhamnose	—	—	—	—	—	—	—	—	—	2498.4 ± 0.8	241.2 ± 0.9	10,148.0 ± 0.8
Glucuronic acid	123.1 ± 0.5	86.9 ± 0.4	85.0 ± 0.9	275.9 ± 0.6	161.0 ± 0.8	66.0 ± 0.4	88.3 ± 0.2	66.0 ± 0.1	143.8 ± 0.6	1186.6 ± 0.4	452.5 ± 0.8	4374.0 ± 0.3
Galacturonic acid	—	—	—	110.7 ± 0.65	—	—	41.5 ± 0.5	—	—	800.7 ± 0.7	135.7 ± 0.4	4128.7 ± 0.8
Glucose	6126.4 ± 0.7	5335.3 ± 0.8	4992.4 ± 0.8	13,067.8 ± 0.9	10,665.7 ± 0.8	4605.5 ± 0.8	6281.8 ± 0.6	4605.5 ± 0.6	7080.2 ± 0.1	19,855.6 ± 0.1	17,016.4 ± 0.1	21,137.7 ± 0.1
Galactose	—	6.9 ± 0.1	5.7 ± 0.1	—	—	—	—	—	—	8929.1 ± 0.8	2109.5 ± 0.7	47,749.9 ± 0.9
Xylose	—	7.0 ± 0.5	0.80 ± 0.15	4.8 ± 0.1	7.7 ± 0.1	14.7 ± 0.1	—	14.7 ± 0.4	—	228.6 ± 0.5	22.6 ± 0.1	355.8 ± 0.9
Arabinose	3.2 ± 0.1	—	—	10.7 ± 0.7	11.7 ± 0.5	5.1 ± 0.1	—	5.1 ± 0.7	—	5877.5 ± 0.5	1512.5 ± 0.1	30,350.2 ± 0.9
Fucose	—	—	—	11.3 ± 0.1	—	—	—	—	—	847.5 ± 0.1	302.8 ± 0.5	1477.4 ± 0.7
Total monosaccharides	6324.9 ± 0.1	5495.6 ± 0.9	5135.9 ± 0.4	13,607.6 ± 0.7	10,931.8 ± 0.1	4733.8 ± 0.5	6469.0 ± 0.3	4703.7 ± 0.6	7235.0 ± 0.3	47,164.1 ± 0.7	23,476.1 ± 0.9	12,7049.6 ± 0.5
Aspartic acid	5.0 ± 0.2	4.0 ± 0.1	4.0 ± 0.1	5.0 ± 0.3	4.0 ± 0.4	—	4.0 ± 0.5	—	—	—	—	—
Glutamic acid	—	—	—	—	—	—	—	—	—	—	—	—
Hydroxyproline	2.0 ± 0.3	1.0. ± 0.1	1.0 ± 0.1	1.0 ± 0.1	1.0 ± 0.1	1.0 ± 0.1	2.0 ± 02	—	—	—	—	—
Serine	—	—	1.0 ± 0.1	—	1.0 ± 0.1	1.0 ± 0.1	—	—	—	—	—	—
Glycine	2.0 ± 0.4	2.0 ± 0.3	2.0 ± 0.2	2.0 ± 0.1	2.0 ± 0.05	2.0 ± 0.3	2.0 ± 0.6	—	—	—	—	—
Histidine	—	1.0. ± 0.4	—	1.0 ± 0.1	1.0 ± 0.2	—	—	—	—	—	—	—
Arginine	—	—	—	—	—	—	—	—	—	—	—	—
Threonine	7.0 ± 0.4	4.0 ± 0.3	5.0 ± 0.8	4.0 ± 0.2	5.0 ± 0.1	3.0. ± 0.2	5.0 ± 0.3	—	—	—	—	—
Alanine	—	1.0. ± 0.1	—	1.0 ± 0.2	1.0 ± 0.2	—	1.0 ± 0.1	—	—	—	—	—
Proline	—	—	—	1.0 ± 0.08	1.0 ± 0.11	1.0 ± 0.05	—	—	—	—	—	—
Lysine	1.0 ± 0.1	1.0 ± 0.2	1.0 ± 0.1	1.0 ± 0.1	1.0 ± 0.1	1.0 ± 0.1	1.0 ± 0.1	—	—	—	—	—
Total amino acids	17.0 ± 0.2	14.0 ± 0.5	13.0 ± 0.8	16.0 ± 0.5	16.0 ± 0.5	9.0 ± 0.5	15.0 ± 0.6	—	—	—	—	—
Oxalic acid	1.0 ± 0.1	0.50 ± 0.01	0.2 ± 0.03	1.40 ± 0.05	0.80 ± 0.08	3.30 ± 0.06	2.90 ± 0.01	0.10 ± 0.01	—	—	—	—
Tartaric acid	109.0 ± 0.8	116.0 ± 0.8	24.0 ± 0.1	53.0 ± 0.1	34.0 ± 0.4	5.0 ± 0.3	4.0 ± 0.6	—	—	—	—	—
Malic acid	—	—	—	11.0 ± 0.5	3.0 ± 0.3	5.0 ± 0.4	4.0 ± 0.6	—	—	—	—	—
Lactic acid	244.0 ± 0.1	188.0 ± 0.4	53.0 ± 0.4	38.0 ± 0.6	57.0 ± 0.4	347.0 ± 0.9	285.0 ± 0.3	47.0 ± 0.6	34.0 ± 0.5	—	—	—
Acetic acid	272.0 ± 0.8	201.0 ± 0.6	45.0 ± 0.9	21.0 ± 0.8	71.0. ± 0.8	64.0 ± 0.2	6.0 ± 0.4	35.0 ± 0.2	20.0 ± 0.6	—	—	—
Maleic acid	—	—	—	—	—	—	—	±0.01	±0.01	—	—	—
Citric acid	56.0 ± 0.9	44.0 ± 0.8	19.0 ± 0.2	436.0 ± 0.3	345.0 ± 0.1	178.0 ± 0.1	157.0 ± 0.6	12.0 ± 0.8	7.0 ± 0.8	—	—	—
Fumaric acid	—	—	—	0.40 ± 0.06	0.30 ± 0.02	0.30 ± 0.06	0.30 ± 0.02	—	—	—	—	—
Succinic acid	49.0 ± 0.6	41.0 ± 0.6	8.0 ± 0.9	12.0 ± 0.2	23.0 ± 0.5	22.0 ± 0.5	20.0 ± 0.2	2.0 ± 0.4	1.0 ± 0.3	—	—	—
Total organic acids	734.0 ± 0.5	594.0. ± 0.9	152.0 ± 0.2	575.0 ± 0.1	537.0 ± 0.5	626.0 ± 0.8	482.0 ± 0.4	97.0 ± 0.6	64.0 ± 0.4	—	—	—

“—” means not detected. SUC (sucrose water); PO (palm oil); BJ (Chinese Baijiu); B (beer); HJ (Chinese Huangjiu); W (Wine); BJD (de-alcoholized Baijiu); BD (de-alcoholized beer); HJD (de-alcoholized Huangjiu); WD (de-alcoholized wine); WDX5 (quintuple dilution of de-alcoholized wine); alcohol (ethanol diluted in distilled water).

## Data Availability

The data presented in this study are available on request from the corresponding author due to privacy.

## Supplementary Materials

The following supporting information can be downloaded at: https://www.mdpi.com/article/10.3390/molecules29081693/s1, Figure S1: Changes in bee survival rate; Figure S2: Total phenol content in different diets of bees; Figure S3: Agarose gel electrophoresis of PCR products; Table S1: Procedure of elution; Table S2: Bee diet formula.
