# Supplementary material for: Effects of Active Ingredients in Alcoholic Beverages and Their De-Alcoholized Counterparts on High-Fat Diet Bees: A Comparative Study
Source: Molecules. 2024 Apr 9;29(8):1693. doi: 10.3390/molecules29081693 (PMC11052269; doi:10.3390/molecules29081693)
Supplement: Supplementary file 1 [file molecules-29-01693-s001.zip › molecules-2932180-supplementary.pdf]

Materials and Methods

1. Reagents and chemicals

In the study, a comprehensive array of reagents and chemicals essential for the experimental procedures was meticulously selected for their chromatographic and analytical grade to ensure the reliability and accuracy of the results. Methanol, ethanol, potassium dihydrogen phosphate, sodium hydroxide, 1-phenyl-3-methyl-5-pyrazolone, hydrochloric acid, chloroform, trifluoroacetic acid, acetonitrile, sodium acetate, disodium hydrogen phosphate, phosphoric acid, phenyl isothiocyanate, triethylamine, hexane, and phenol were sourced from Shanghai Macklin Biochemical Co., Ltd., affirming their chromatographic grade suitability for the intended analytical applications. Further, specific phenolic compounds such as gallic acid, vanillic acid, coumaric acid, and p-coumaric acid were acquired from Shanghai Yuanye Bio-Technology Co., Ltd. Ferulic acid was procured from Chengdu Desite Biological Technology Co., Ltd. Additionally, a range of compounds including sucrose, caffeic acid, catechin, epicatechin, quercetin, gallic acid monohydrate, chlorogenic acid, rutin, and p-hydroxybenzoic acid were purchased from Beijing Solarbio Science & Technology Co., Ltd. Moreover, sodium carbonate anhydrous, Folin-Ciocalteu reagent, mustard acid, 3,4-dihydroxybenzoic acid, mannose, ribose, rhamnose, glucuronic acid, galacturonic acid, N-acetyl-glucosamine, glucose, N-acetyl-galactosamine, galactose, xylose, arabinose, fucose, aspartic acid, glutamic acid, serine, glycine, histidine, arginine, threonine, alanine, proline, cysteine, ammonium chloride, tyrosine, valine, methionine, isoleucine, leucine, phenylalanine, lysine, oxalic acid, tartaric acid, malic acid, lactic acid, acetic acid, citric acid, fumaric acid, and succinic acid were also obtained from Shanghai Macklin Biochemical Co., Ltd., highlighting the procurement of analytical grade reagents to ensure the high standard of chemical analyses conducted within the research. All of the above reagent companies are from China. Palm oil, utilized as part of the experimental materials, was procured from Polydragon Holiday Palm Oil (Indonesia).

2. Alcoholic beverages and their de-alcoholized counterparts and bee diet composition analysis

2.1 The quantification of monosaccharides

In the described study, accurate measurements of alcoholic beverages, their non-alcoholic counterparts, and bee diet were taken and placed into 5 mL ampoules. To each of these ampoules, 5.0 mL of 2 M trifluoroacetic acid (TFA) was added, after which the vials were sealed and subjected to acid hydrolysis at a temperature of 110°C for a duration of 8 hours. Following the hydrolysis process, the ampoules were removed from the heat source to allow the TFA to evaporate completely. The resulting residues were then redissolved in 5.0 mL of water. For the purpose of creating a mixed standard solution, precise quantities of 12 monosaccharide standards were weighed and dissolved in water to achieve a concentration of 50 μg/mL.

The next phase involved the preparation of a derivatized standard solution for High-Performance Liquid Chromatography (HPLC) (Waters, Milford, MA, USA) analysis. This entailed the accurate transfer of 250 μL of the mixed standard solution into a 5 mL centrifuge tube, followed by the addition of 250 μL of 0.6 M NaOH and 500 μL of 0.4 M 1-phenyl-3-methyl-5-pyrazolone (PMP) in methanol. This mixture was then incubated at 70°C for 1 hour to facilitate the reaction, after which it was cooled in cold water for 10 minutes. The reaction was neutralized

by adding 500 μL of 0.3 M HCl, followed by the addition of 1 mL of chloroform. The mixture underwent vortexing for 1 minute and was then centrifuged at 3000 rpm for 10 minutes. The supernatant was meticulously collected, and the extraction process was repeated thrice. The final aqueous phase was filtered through a 0.22 μm organic membrane to prepare it for subsequent HPLC analysis. The sample derivatization solution was prepared following the identical steps used for the standard solution.

For the analytical procedure, an Xtimate C18 column (4.6 x 200 mm, 5 μm) (Waters, Milford, MA, USA) was employed, with the column temperature regulated at 30°C. The volume of each injection was set at 20 μL, with a flow rate maintained at 1.0 mL/min. Detection of the analytes was carried out at a wavelength of 250 nm (Refractive Index Detector, RID). (Waters, Milford, MA, USA). The mobile phase used for this analysis comprised a 0.05 M solution of potassium dihydrogen phosphate, the pH of which was adjusted to 6.70 using a sodium hydroxide solution, mixed with acetonitrile in a ratio of 83:17.

2.2 The quantification of organic acids

Nine organic acids standards were precisely weighed and diluted to the required concentration gradients utilizing the mobile phase. All samples, including standard solutions, alcoholic beverages, their de-alcoholized counterparts, and bee feed, underwent filtration through 0.22-μm organic filters to ensure purity and consistency in the subsequent analysis.

For the analysis, an Xtimate C18 column (4.6 x 200 mm, 5 μm) (Waters, Milford, MA, USA) was used, with the column temperature set at 30°C. The volume of each sample injected into the chromatograph was standardized at 10 μL, with a consistent flow rate of 1.0 mL/min to ensure reproducibility of the results. Detection of the analytes was performed at a wavelength of 210 nm (Refractive Index Detector, RID) (Waters, Milford, MA, USA). The mobile phase used in the chromatographic separation consisted of 50 mM disodium hydrogen phosphate, the pH of which was precisely adjusted to 2.50 using phosphoric acid to achieve optimal separation efficiency and peak resolution.

2.3 The quantification of amino acids

In the experimental procedure detailed within the study, phenyl isothiocyanate and triethylamine, serving as derivatization reagents, were diluted with a suitable diluent to one-fifth of their original concentrations. The analytical sample was accurately weighed and placed into an ampule, to which a 6 mol/L hydrochloric acid solution containing 0.1% phenol (5.0 mL) was added. The ampule was then flushed with nitrogen and sealed securely. This setup was subjected to hydrolysis in an oven maintained at 110°C for a duration of 24 hours. Following hydrolysis, the hydrochloric acid was evaporated under a nitrogen atmosphere, and the residue was redissolved in 5.0 mL of water.

To a 200 μL aliquot of this solution, 100 μL of diluted Solution phenyl isothiocyanate and 100 μL of diluted Solution triethylamine were added, thoroughly mixed, and allowed to react at room temperature for 60 minutes. Subsequently, 400 μL of hexane was added, the cap was secured tightly, and the mixture was vigorously shaken for 5-10 seconds. This mixture was then left to stand at room temperature to facilitate phase separation. Afterward, 200 μL was carefully extracted from the lower layer and combined with 800 μL of water. This diluted solution, now 200 μL, was further mixed with an additional 800 μL of water in preparation for analysis. The amino acid standards, with the exception of hydroxyproline and leucine which were prepared to a concentration of 1 mg/mL, were prepared to a concentration of 2.5 mM. A 200 μL volume of these

standard solutions was accurately measured into a test tube, to which 50 μL of leucine and hydroxyproline standard solutions were added, followed by 100 μL each of diluted Solution phenyl isothiocyanate and Solution triethylamine. This mixture was well blended and allowed to react at room temperature for 60 minutes. Following this, 400 μL of hexane was added, the cap was secured, and the tube was shaken vigorously for 5-10 seconds, allowing for phase separation. A 200 μL sample was extracted from the lower layer, thoroughly mixed with 800 μL of water, and then this mixture was further diluted with water to prepare it for analysis. All samples were filtered through a 0.22 μm organic membrane filter before use.

For chromatographic analysis, an Xtimate C18 column (4.6 x 200 mm, 5 μm) (Waters, Milford, MA, USA) was utilized, with the column temperature regulated at 40°C. An injection volume of 10 μL was chosen, and a consistent flow rate of 1.0 mL/min was maintained throughout. Detection of analytes was performed at a wavelength of 254 nm (Refractive Index Detector, RID). (Waters, Milford, MA, USA), employing a mobile phase that included 50 mM disodium hydrogen phosphate adjusted to pH 2.50 using phosphoric acid. The mobile phase was composed of two solutions: Mobile Phase A, which was a 0.1 mol/L sodium acetate solution adjusted to pH 6.50 and mixed with acetonitrile in a 93:7 ratio, and Mobile Phase B, consisting of a mixture of water and acetonitrile in a 20:80 ratio. The elution procedure is as follows:

Table S1 Procedure of elution

Time（min）	Flow rate（mL/min）	A%	B%
0.01	1	100	0
11	1	93	7
13.9	1	88	12
14	1	85	15
29	1	66	70
32	1	30	100
35	1	0	34
42	1	0	100
45	1	100	0
60	1	100	0

Supplement figures

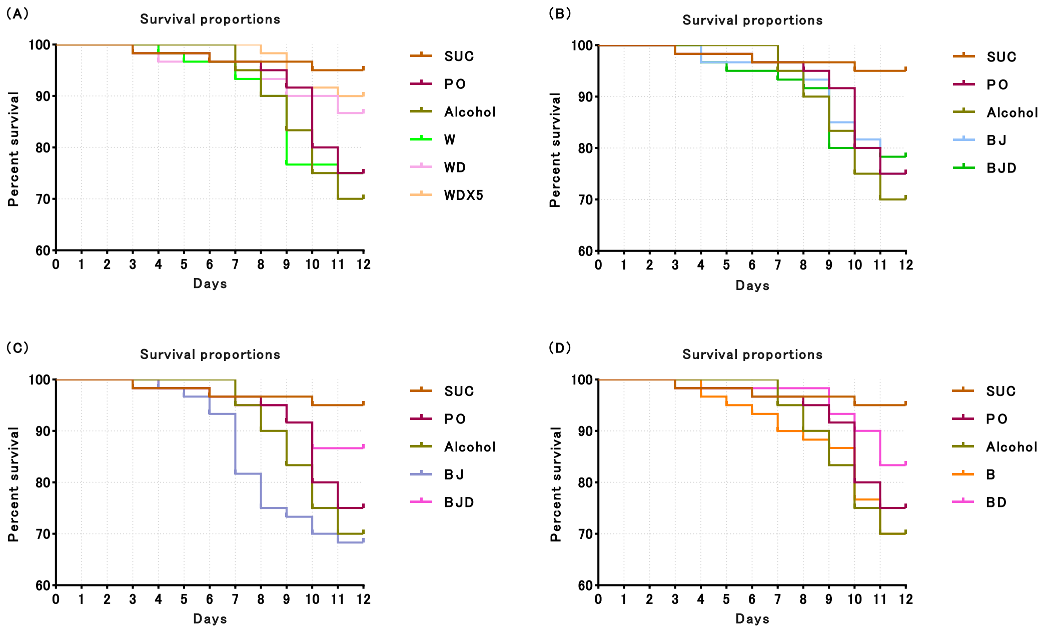

Figure S1. Changes in bee survival rate. Sugar water treatment group (SUC), palm oil treatment group (PO), alcohol treatment group (Alcohol), Baijiu/de-alcoholized Baijiu treatment group (BJ/BJD), Huangjiu/de-alcoholized Huangjiu treatment group (HJ/HJD), beer/de-alcoholized beer treatment group (B/BD), wine/de-alcoholized wine /de-alcoholized wine diluted fivefold treatment group (W/WD/WDX5)

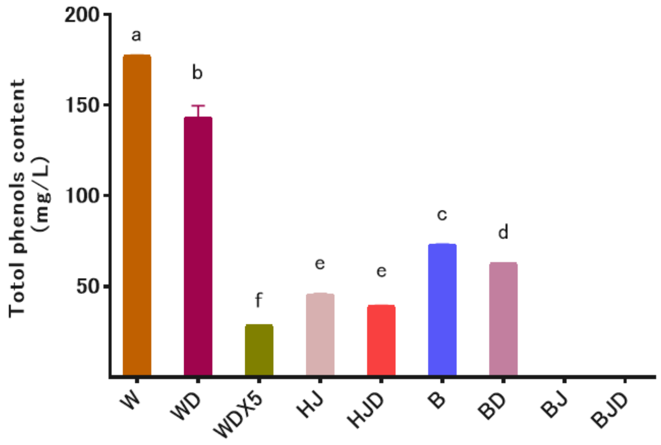

Figure S2. Total phenol content in different diets of bees. Baijiu/de-alcoholized Baijiu treatment group (BJ/BJD), Huangjiu/de-alcoholized Huangjiu treatment group (HJ/HJD), beer/de-alcoholized beer treatment group (B/BD), wine/de-alcoholized wine /de-alcoholized wine diluted fivefold treatment group (W/WD/WDX5), different letters in the graph indicate significant differences between data, P< 0.05.

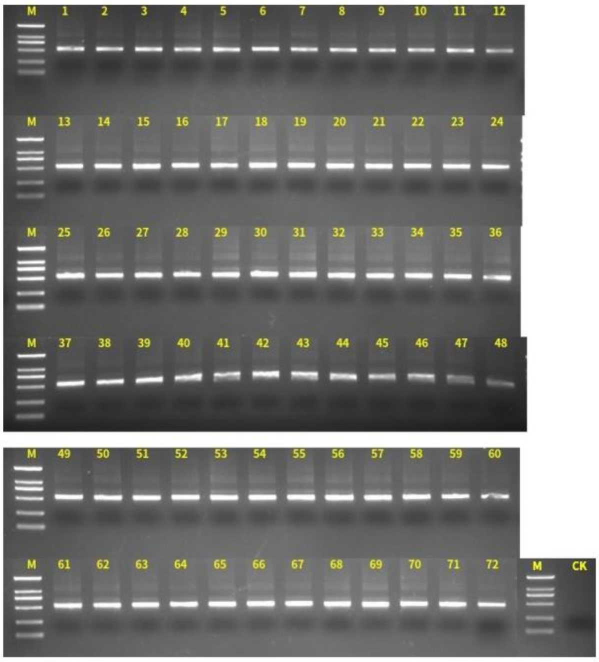

Figure S3. Agarose gel electrophoresis of PCR products. Sugar water treatment group (SUC), palm oil treatment group (PO), alcohol treatment group (Alcohol), Baijiu/de-alcoholized Baijiu treatment group (BJ/BJD), Huangjiu/de-alcoholized Huangjiu treatment group (HJ/HJD), beer/de-alcoholized beer treatment group (B/BD), wine/de-alcoholized wine /de-alcoholized wine diluted fivefold treatment group (W/WD/WDX5), 1 to 6 for SUC1 to SUC6; 7 to 12 for PO1 to PO6; 13 to 18 for Alcohol1 to Alcohol6; 19 to 24 for W1 to W6; 25 to 30 for WD1 to WD6; 31 to 36 for WD1X5 to WD6X5; 37 to 42 for B1 to B6; 43 to 48 for BD1 to BD6; 49 to 54 for HJ1 to HJ6; 55 to 60 for HJD1 to HJD6; 61 to 66 for BJ1 to BJ6; 67 to 72 for BJD1 to BJD6.

Table S2 Bee diet formula

Group	Diet
SUC	50% SUC
PO	2% PO+50% SUC
Alcohol	2% PO+50% SUC + 1% Alcohol
BJ	2% PO+50% SUC + 1% Baijiu
B	2% PO+50% SUC + 1% Beer
HJ	2% PO+50% SUC + 1% Huangjiu
W	2% PO+50% SUC + 1% Wine
BJD	2% PO+50% SUC + 1% de-alcoholized Baijiu
BD	2% PO+50% SUC + 1% de-alcoholized beer
HJD	2% PO+50% SUC + 1% de-alcoholized Huangjiu
WD	2% PO+50% SUC + 1% de-alcoholized wine
WDX	2% PO+50% SUC + 1% de-alcoholized wine diluted fivefold

Sugar water treatment group (SUC), palm oil treatment group (PO), alcohol treatment group (Alcohol), Baijiu/de-alcoholized Baijiu treatment group (BJ/BJD), Huangjiu/de-alcoholized Huangjiu treatment group (HJ/HJD), beer/de-alcoholized beer treatment group (B/BD), wine/de-alcoholized wine /de-alcoholized wine diluted fivefold treatment group (W/WD/WDX5)
